# Fabrication of Hollow Channels Surrounded by Gold Nanoparticles in Hydrogel by Femtosecond Laser Irradiation

**DOI:** 10.3390/nano10122529

**Published:** 2020-12-16

**Authors:** Izumi Takayama, Akito Katayama, Mitsuhiro Terakawa

**Affiliations:** 1 School of Integrated Design Engineering, Keio University, 3-14-1, Hiyoshi, Kohoku-ku, Yokohama 223-8522, Japan; i.takayama@tera.elec.keio.ac.jp (I.T.); akito.32.kata@gmail.com (A.K.); 2 Department of Electronics and Electrical Engineering, Keio University, 3-14-1, Hiyoshi, Kohoku-ku, Yokohama 223-8522, Japan

**Keywords:** femtosecond laser, PEGDA, multiphoton reduction, gold nanoparticles, hollow channel

## Abstract

The fabrication of hollow channels surrounded by gold nanoparticles in poly(ethylene glycol) diacrylate (PEGDA) is demonstrated. The absorption spectra show that gold nanoparticles were formed at the periphery of the focus by reduction of gold ions. The microscope observation and Raman spectroscopy analyses indicate that the center of the channels were void of PEGDA, which can be attributed to the femtosecond laser-induced degradation of the hydrogel. Since both the hydrogel and gold nanoparticles are biocompatible, this technique of fabricating hollow channels surrounded by gold nanoparticles is promising for tissue engineering, drug screening, and lab-on-a-chip devices.

## 1. Introduction

The fabrication of tissue in vitro is essential in advancing the development of tissue engineering and drug screening. A tissue scaffold is necessary for growing cells and to support the tissue. Cell adhesion, differentiation, and proliferation are highly dependent on cell interactions with the tissue scaffold; therefore, it is necessary to control the shape and properties of the tissue scaffold when fabricating a tissue that resembles biological tissue. The mechanical strength of the material of the tissue scaffold should be comparable to that of the tissue from which the cell originates [1]. In addition, a chemical composition and surface structure that has a high compatibility to cells are required for the tissue scaffold.

Hydrogels have been used as tissue scaffolds because of their high biocompatibility. Because the mechanical strength of hydrogels can be tuned by the water content and molecular weight, hydrogels have been utilized as scaffolds for soft tissues, such as nerve tissue, to harder tissues, such as bone tissue [2]. In addition, hydrogels show high permeability to liquid, which allows the diffusion of glucose, oxygen and other nutrients to cells adhered in the bulk hydrogel [3]. 3-dimensional tissues fabricated on hydrogel tissue scaffolds have applications in tissue engineering including the regeneration of spinal cords, but also can be useful in organ-on-a-chip, drug screening, and diagnostics [4,5,6,7,8]. Existing methods for processing bulk hydrogel for the fabrication of tissue scaffolds include, multiphoton polymerization, soft lithography, molding using sacrificial materials, and laser processing [9]. Femtosecond lasers are powerful tools for the fabrication of a 3-dimensional channel in bulk materials including hydrogels. Liu et al. fabricated a channel in collagen and showed the adhesion of human HT1080 fibroblasts aligned along the channels [10]. Sarig-Nadir et al. fabricated channels, with 4–17 µm width and 10–80 µm height, in a bulk poly(ethylene glycol) diacrylate (PEGDA) based hydrogel, one of the most typical hydrogels, by irradiating femtosecond or nanosecond laser pulses [11]. Dorsal root ganglion cells adhered in the channels and aligned along the direction of the channel. Because PEGDA does not induce high cell adhesion on its own, PEGDA mixed with fibrinogen cysteines was used for the tissue scaffold.

Gold nanoparticles have a high biocompatibility and allow the adsorption of proteins which enhance cell adhesion [12]. In recent years, it has been reported that the patterning of gold nanoparticles on the surface of hydrogels accelerates cell adhesion in monolayer cultures. Ren et al. patterned 10 µm wide arrays consisting of 20 nm gold nanoparticles on the surface of PEGDA hydrogels by microcontact printing. Murine fibroblast L-929 cells were reported to have only adhered to the region with patterned gold nanoparticles [13].

It has been reported that by focusing femtosecond laser pulses into the bulk of a material that contains metal ions, metal particles generated by photoreduction dispersed around the focal point. Tosa et al. irradiated a femtosecond laser pulse at a repetition rate of 82 MHz into a film consisting of gold chloride and poly (4-styrene sulfonic acid) [14]. In the fabricated structure, no gold nanoparticles were observed at the center of the focus but were precipitated around the focal point, which were considered to be attributable to the gradient of the electric field to thrust the nanoparticles to the edge of the laser beam. Bellec et al., used a femtosecond laser pulse at a repetition rate of 9.44 MHz to fabricate a silver structure by irradiating the pulse into silver doped phosphate glass [15]. The resulting structure consisted of clusters of silver nanoparticles at the edge of the laser beam. The photodissociation of silver nanoparticles in the focal point were discussed to explain the distribution. To the best of our knowledge, the simultaneous fabrication of hollow channels and gold nanoparticles around the channels by laser irradiation has yet to be reported.

In this paper, we demonstrate the simultaneous fabrication of hollow channels surrounded by gold nanoparticles in hydrogels by femtosecond laser irradiation. The bright-field microscope images and absorption spectra show the reduction of gold ions around the channels, induced by femtosecond laser irradiation. In addition, the digital microscope images and Raman spectroscopy results indicate that the center of the channels were void of PEGDA matrix.

## 2. Materials and Methods

### 2.1. Hydrogel Preparation

Hydrogels were prepared by dissolving 0.1 g of PEGDA (molecular weight 4000) (Polysciences, Warrington, PA, USA) in 1 mL of pure water, containing 1% Irgacure 2959 initiator (Sigma-Aldrich Co. LLC, St. Louis, MI, USA). The PEGDA solution was stirred with a magnetic stirrer for 30 min until completely dissolved. The solution was added to a silicon mold with dimensions of 8 mm × 11 mm × 2.5 mm. The PEGDA solution was irradiated with an ultraviolet (UV) lamp (LUV-16, AXEL, Osaka, Japan) with a central wavelength of 365 nm for 1 h to induce photo-crosslinking. The crosslinked hydrogels were immersed in a gold chloride solution (Fujifilm Wako Chemicals, Osaka, Japan) for 10 min to induce the uptake of gold ions into the hydrogel matrix. The concentrations of gold chloride (AuCl_4_) solution used in this paper was 1, 2, 4, or 8 mg/mL. After immersing the fabricated hydrogels in water for 1 day, black India ink was injected into the channels with a syringe (Terumo, Tokyo, Japan) to assess whether the channels are hollow. The channels with black India ink, were observed with a bright-field microscope.

### 2.2. Laser Parameters

**Figure 1:**
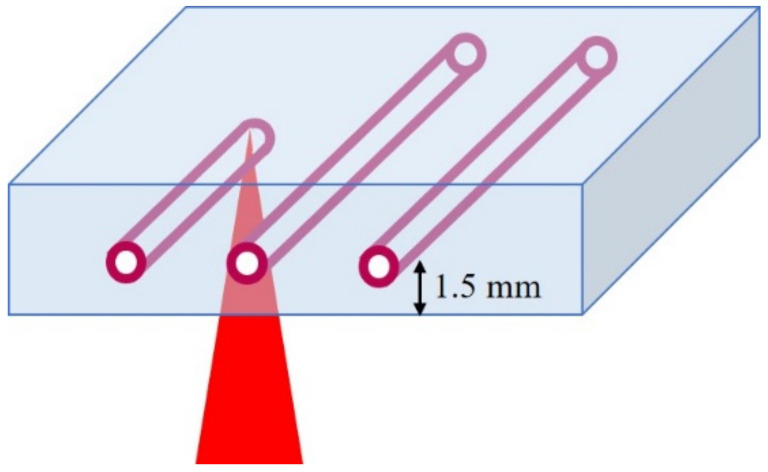
Schematic diagram of the simultaneous fabrication of hollow channels and surrounding gold nanoparticles.

A Ti:sapphire chirped pulse amplification (CPA) laser system (Libra, Coherent. Inc., Santa Clara CA, USA) generated linearly polarized femtosecond laser pulses with a pulse duration of 100 fs, at a central wavelength of 800 nm, at a repetition rate of 1 kHz. PEGDA hydrogels were placed onto a glass slide on a XY stage. Femtosecond laser pulses were focused into PEGDA hydrogels with an objective lens (60×, NA 0.7). The setup of the experiment is shown in Figure 1. In hydrogels that were immersed in gold chloride solution, all structures were fabricated with a scanning speed of 200 µm/s and 2 scans. In order to prevent the shrinkage of the hydrogel while fabricating channels, the same concentration of gold chloride solution that the hydrogel was immersed in, was dropped on the hydrogel. Pulse energies of 2–10 µJ and gold chloride concentrations of 1–8 mg/mL were used to fabricate structures in PEGDA hydrogels. The channels were observed with a bright-field microscope (Eclipse Ti-E, Nikon, Tokyo, Japan). The hydrogels were cut by an iron gel cutter to observe the cross-sections of the hydrogels. The widths of the channels were measured from the bright-field microscope images of the top view of the channels by using ImageJ. For the fabrication of a channel with a larger width, a structure consisting of 3 parallel channels at an interval of 50 µm was fabricated at a pulse energy of 10 µJ and gold chloride solution concentration of 4 mg/mL.

### 2.3. Absorption Spectrum Measurements

The absorption spectra of the channels were measured to evaluate the relative concentration of gold nanoparticles formed around the channels. A white light was irradiated onto gratings consisting of channels inside a hydrogel. The transmission light was coupled to an optic fiber connected to an ultraviolet-visible-near infrared spectrophotometer (USB4000, Ocean Optics, Largo, FL, USA). A glass slide was used for the background correction. 

### 2.4. Digital Microscope

A digital microscope (HRX-01, HIROX, Tokyo, Japan) was used to acquire the bright-field images of the cross-sectional view of the fabricated structures. Both the 2-dimensional and 2.5-dimensional image of the cross-sectional view of the structures were obtained.

### 2.5. Raman Spectroscopy

A laser-excited Raman spectrometer (InVia Raman Microscope, Renishaw, Wotton-under-Edge, UK) was used to analyze the chemistry of the fabricated structures. The excitation wavelength was 532 nm and the structures were analyzed for a wavenumber range of 100 to 3000 cm^−1^. A 20× objective lens (NA 0.40) was used to focus the laser at the center of the cross-section of the structures and the unirradiated region of the hydrogel. From the wavelength and NA, the spatial resolution is estimated to be approximately 1.6 µm.

## 3. Results and Discussion

### 3.1. Fabrication of Hollow Channels Surrounded by Gold Nanoparticles

**Figure 2:**
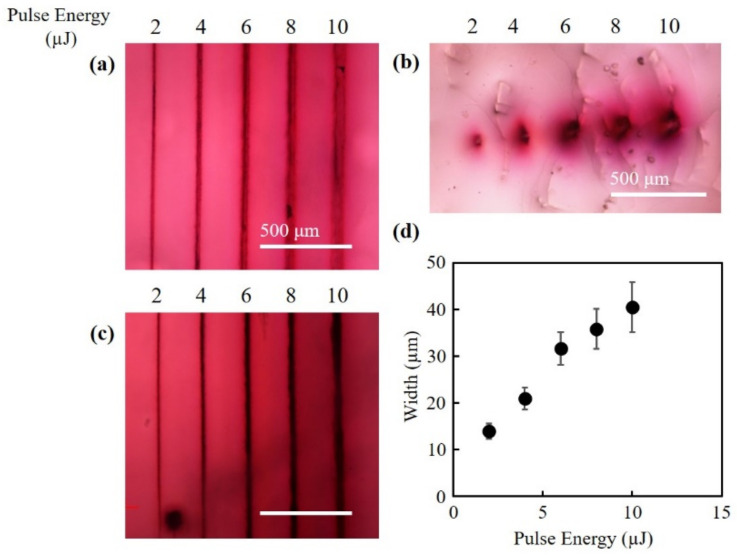
Fabrication of channels at different pulse energies. The bright-field microscope images of the (**a**) top view and (**b**) cross-sectional view of the fabricated channels. (**c**) The bright-field microscope images of the channels with ink. (**d**) The widths of the channels fabricated at different pulse energies.

Figure 2a–c shows the bright-field microscope images of the structures fabricated at pulse energies of 2–10 µJ, a scan speed of 200 µm/s, and 2 scans. The concentrations of the gold chloride solution was 4 mg/mL for all structures. The top view and cross-sectional view of the fabricated structures are shown in Figure 2a–c, respectively. By immersing the hydrogel in gold chloride solution, a spontaneous color change from transparent to reddish color was observed in the hydrogel with fabricated channels, where the laser pulse was not irradiated, similar to the case of other studies using gold chloride solution [16]. In the cross-sectional view of the structures (Figure 2b), the change in color at the center of the structures was small, whereas the change in color around the structures was significant. The color of the structures changed from a light red to a darker red with the increase of pulse energy, suggesting that the density of the gold nanoparticles increased. As indicated in Figure 2c, the injected ink flowed into the fabricated channels. These results suggest that a hollow channel with gold nanoparticles distributed around the channel was fabricated. Figure 2d shows the relationship between the pulse energy and the widths of the fabricated channels. The widths of the channels increased with the increase in pulse energy. Even with the lowest pulse energy in the present study, 2 µJ, the intensity of the laser pulse was calculated to be 1.71 × 10^15^ W/cm^2^, which is significantly higher than the reported threshold of the optical breakdown in water, which is in the range of 10^13^ W/cm^2^ [17,18]. Therefore, it is highly probable that the laser induced plasma contributed to the fabrication of the structures.

**Figure 3:**
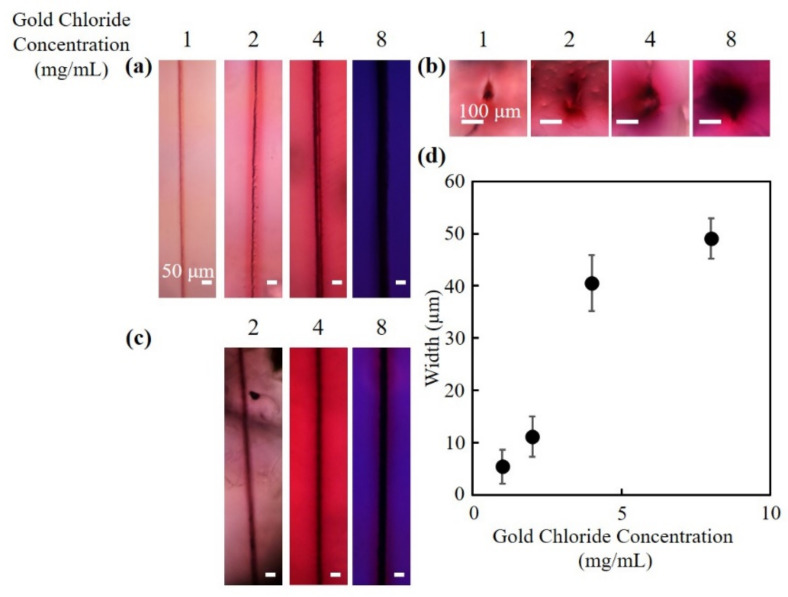
Fabrication of channels at different gold chloride concentrations. The bright-field microscope images of the (**a**) top view and (**b**) cross-sectional view of the fabricated channels. (**c**) The bright-field microscope images of the channels with ink. (**d**) The widths of the channels fabricated at different gold chloride concentrations.

Figure 3a,b show the top view and cross-sectional view of the structures fabricated at different concentrations of gold chloride solutions, respectively. All structures were fabricated at a pulse energy of 10 µJ, a scan speed of 200 µm/s and 2 scans. In hydrogels that were immersed in gold chloride solution concentrations of 1, 2, 4 mg/mL, the regions where there was no laser irradiations exhibited a reddish color. The color became darker with the increase of the gold chloride solution concentration. However, in hydrogels that were immersed in a gold chloride solution with a concentration of 8 mg/mL, the color of the unirradiated hydrogel was a bluish purple. The discussions for the result will be described in the next section. In hydrogels that were immersed in gold chloride solution concentration of 1 mg/mL, ink did not penetrate the channels. From Figure 3c, it can be observed that the ink penetrated the fabricated structures at gold chloride solution concentrations of 2, 4, and 8 mg/mL. The results suggest that hollow channels were fabricated, similar to Figure 2c. The widths of the channels increased with the increase in gold chloride solution concentration (Figure 3d). It is thought that in hydrogels that were immersed in higher concentrations of gold chloride solution, more gold nanoparticles were generated spontaneously or by photoreduction with primary laser pulses. The widths of the channels probably increased due to the change of absorption coefficient of the hydrogel by the nanoparticle generation. When a laser pulse with an intensity above the threshold of the optical breakdown of water is irradiated into a hydrogel, electrons in the water are excited in a short time, which results in the generation of plasma at the focus of the laser pulse. Because the water in the focus flows outward, the pressure at the focus becomes exceedingly low, and consequently the water undergoes a phase change from water to vapor, which results in the generation of a cavitation bubble [19]. As the cavitation bubble expands, the polymer bonds of the hydrogel would be physically dissociated, leading to hydrogel degradation. The dissociation of the bonds and the generation of shockwave are probably the mechanisms behind the fabrication of hollow channels in hydrogels [20]. The gold nanoparticles surrounding the channels were formed by the reduction of gold ions. It has been reported that, by inducing multiphoton reduction with the irradiation of a femtosecond laser pulse, metal ions have been reduced into metal nanoparticles in a hydrogel [16,21,22]. The reduction of metal ions is attributable to the electron donation induced through multiphoton absorption (multiphoton reduction) and/or thermal reduction from the heat accumulation caused by high repetition rates laser pulse. The photoreduction of metal ions can also be induced by radicals from photoinitiator. Izquierdo-Lorenzo et al. used biphenyl-(2,4,6-trimethylbenzoyl)-phosphine oxide (TPO) as a photoinitator to fabricate gold nanoparticles by two-photon photoreduction [23]. In addition, the reduction of metal ions can be induced by a plasma produced by a focused laser pulse. Tasche et al. discussed that the electrons at the boundary of the plasma and silver nitrate solution reduced the silver ions to create silver nanoparticles [24]. The gold nanoparticles in the structures formed in the present study were distributed to regions larger than the focal spot size of the laser pulse. As described above, the laser intensities in the present study were higher than the threshold of the optical breakdown in water, which could result in the generation of plasma. Possible explanations are that either the gold nanoparticles generated by multiphoton reduction were pushed to the periphery by the expansion of the plasma and subsequent shockwave, or that the gold ions were reduced by plasma ion radiation at the interface of plasma and hydrogel.

### 3.2. Absorption Spectra of the Fabricated Channels

**Figure 4:**
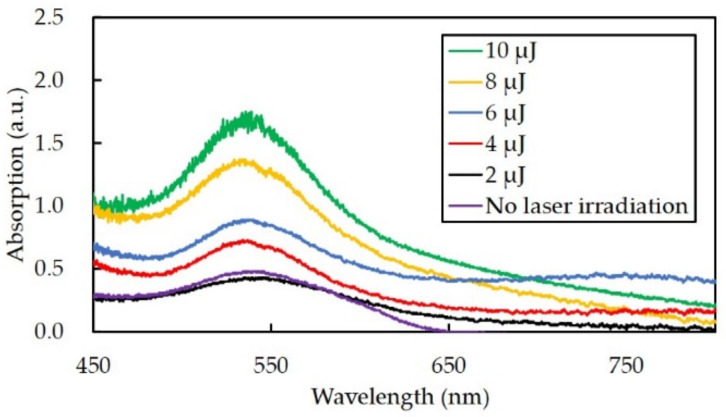
The absorption spectrum of channels fabricated at different pulse energies.

In order to measure the relative density of the gold nanoparticles, the absorption spectrum of the structures was obtained. The absorption spectrum of the structures that were fabricated with a pulse energy of 2–10 µJ, a scan speed of 200 µm/s and 2 scans are shown in Figure 4. The increase in pulse energy led to an increase in the peak height of the absorption spectrum. The results suggest that the number of formed gold nanoparticles increased with the increase of pulse energy. In addition, the absorbance peak was approximately 540 nm, which corresponds to the resonance wavelength of gold nanoparticles. The diameter of the gold nanoparticles was calculated from the equation below, where *λ_SPR_* is the absorbance peak [25].

(1) $$d=\frac{\ln\left(\frac{\lambda_{SPR}-512}{0.0216}\right)}{6.53}$$

From Equation (1), the diameter of the gold nanoparticles was calculated to be approximately 67 nm. In previous studies, we have reported that the diameter of gold nanoparticles fabricated by multiphoton photoreduction was approximately 10 nm [16]. From the unaltered color transmission electron microscopy (TEM) images in our previous studies, it is thought that the calculated diameter in the present study is probably the value for the gold nanoparticle in the form of aggregation [16].

**Figure 5:**
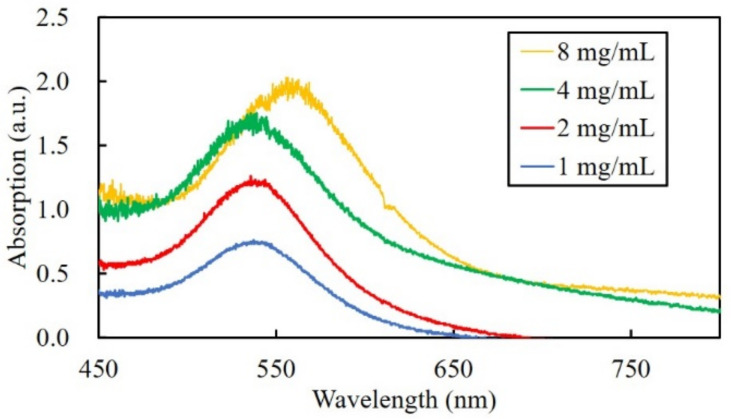
The absorption spectrum of channels fabricated at different gold chloride concentrations.

Figure 5 shows the absorption spectrum of the channels fabricated in hydrogels with different gold chloride solution concentrations. The peak height increased with the increase of gold chloride solution concentration, which indicates that the number of gold nanoparticles increased. In gold chloride solution concentrations of less than and including 4 mg/mL, the absorbance peak of the fabricated channels was approximately 540 nm. However, in gold chloride solution concentrations of 8 mg/mL, the absorbance peak of the channels was approximately 560 nm. The diameter of the gold cluster calculated from the absorbance wavelength was estimated to be 92 nm. The result suggests that the size of the gold nanoparticles, probably in the aggregated form, increased at a high concentration of gold chloride solution. The red shift of the absorption peak corresponds to the results shown in Figure 3, in which the color of the hydrogel changed to bluish purple. In multiphoton photoreduction, subsequent laser pulses irradiated to the gold nanoparticles would induce growth and aggregation of nanoparticles due to the plasmonic-enhanced photoreduction and photothermal effect. The results suggest that when the concentration of gold chloride solution was high, the gold ion in the vicinity of the laser focus was not depleted, which led to the formation of nanoparticles with a larger diameter.

### 3.3. Fabrication of Structures with Different Number of Pulses

**Figure 6:**
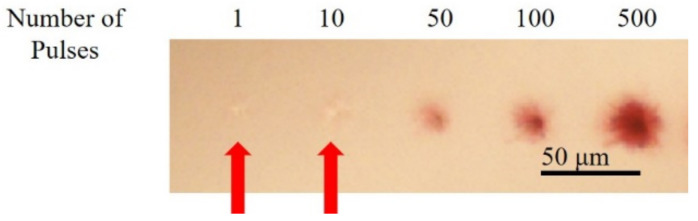
The bright-field microscope images of structures fabricated by irradiating a different number of pulses. The arrows indicate the modification of hydrogel, by laser irradiation, without significant color change.

To investigate the interactions between subsequent laser pulses and gold nanoparticles, gold nanoparticles were formed with a different number of laser pulses. The laser pulse was irradiated in a setup similar to the setup shown in Figure 1, but without the laser scanning. The laser pulses at a pulse energy of 10 μJ were irradiated into the hydrogel with a gold chloride concentration of 4 mg/mL. Figure 6 shows that when 1 and 10 pulses were irradiated, modification of the hydrogel in the focal spot can be observed while no identifiable color change was observed. In contrast, when 50 or more laser pulses were irradiated, the color of the irradiated region changed to red. The region where color change was observed expanded with the increase of irradiated laser pulses. Since the repetition rate of the laser pulses was 1 kHz in the present study, heat accumulation by succeeding laser pulses may be negligible. The expansion of the region may be attributed to the absorption of subsequent laser pulses to the gold nanoparticles formed by primary laser pulses.

### 3.4. Anaylsis of Structures Consisting of Parallel Channels

**Figure 7:**
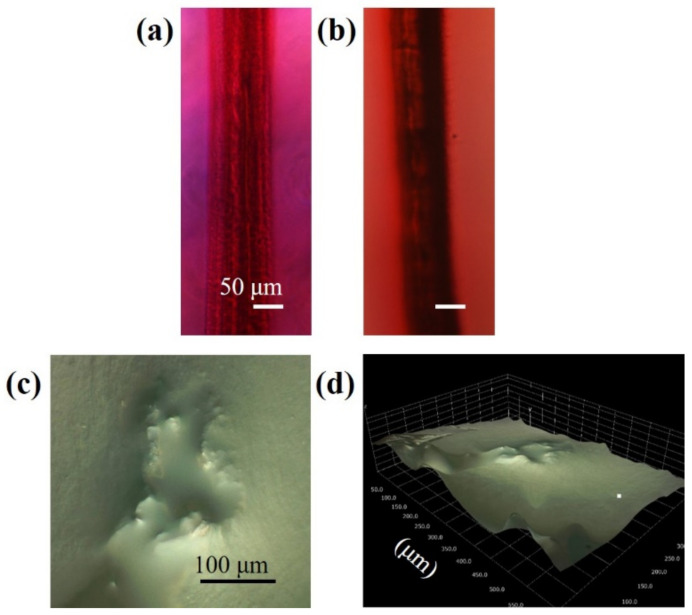
Fabrication of wide channels with applications in tissue engineering, microfluidics, and drug delivery. The bright-field microscope images of the fabricated channels (**a**) without ink and (**b**) with ink. The (**c**) 2-dimensional and (**d**) 2.5-dimensional image of the cross-sectional view of the channels captured by a digital microscope.

Figure 7a shows the bright-field microscope image of structures consisting of 3 parallel channels. The color of the irradiated regions is dark red, which indicates that a significant number of gold ions were reduced by laser irradiation. Black ink penetrated the structure as shown in Figure 7b. In the case of structures consisting of parallel channels, the ink penetrated the structure easily without the use of a syringe. Figure 7c,d show the 2-dimensional and 2.5-dimensionnal images of the cross-sectional view of the structures taken by a digital microscope. The hydrogel was cut perpendicularly to the structures for the observation. The focal point of the microscope did not coincide for the center of the structure and the periphery, which supports the result that a hollow channel was fabricated by the material removal.

**Figure 8:**
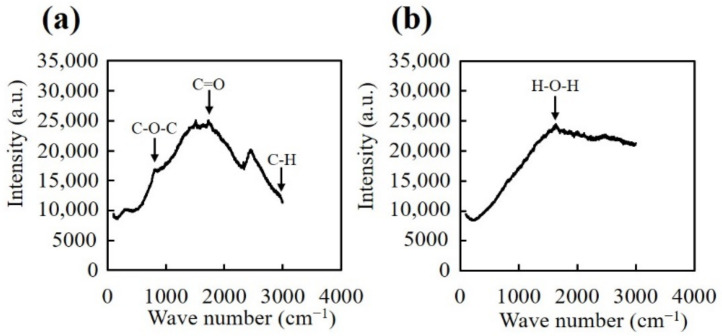
Raman spectra of (**a**) the bulk hydrogel and (**b**) the center of the structures.

Figure 8 shows the result of Raman spectroscopy analyses for the structures consisting of 3 parallel channels. The hydrogel was cut perpendicularly, then the unirradiated region and the center of the structure were analyzed. In unirradiated regions of the hydrogel, the spectrum peaks corresponding to C–O–C (850 cm^−1^), C=O (1720 cm^−1^), and C–H (2950 cm^−1^), respectively were observed, indicating the presence of PEGDA. No peaks corresponding to bonds present in PEGDA were observed at the center of the structures, and only a single peak at 1650 cm^−1^, corresponding to H–O–H bonds was observed. These results also indicate that the center of the structures was void, i.e., without PEGDA matrix.

## 4. Conclusions

In conclusion, the structures consisted of hollow channels which were surrounded by gold nanoparticles. The hollow channels and gold nanoparticles were formed in a single step of irradiating a femtosecond laser pulse in PEGDA hydrogels which contained gold ions. To the best of our knowledge, this is the first example of fabricating a hollow channel surrounded by gold nanoparticles in a hydrogel, by femtosecond laser irradiation. Given that hydrogels and gold nanoparticles have a high biocompatibility, this research has many applications in tissue engineering, microfluidics, and drug delivery.

## References

[1] Butcher D.T., Alliston T., Weaver V.M. (2009). A tense situation: Forcing tumour progression. Nat. Rev. Cancer.

[2] Engler A.J., Sen S., Sweeney H.L., Discher D.E. (2006). Matrix Elasticity Directs Stem Cell Lineage Specification. Cell.

[3] Cruise G.M., Scharp D.S., Hubbell J.A. (1998). Characterization of permeability and network structure of interfacially photopolymerized poly(ethylene glycol) diacrylate hydrogels. Biomaterials.

[4] Chen M.B., Srigunapalan S., Wheeler A.R., Simmons C.A. (2013). A 3D microfluidic platform incorporating methacrylated gelatin hydrogels to study physiological cardiovascular cell-cell interactions. Lab Chip.

[5] Cuchiara M.P., Allen A.C.B., Chen T.M., Miller J.S., West J.L. (2010). Multilayer microfluidic PEGDA hydrogels. Biomaterials.

[6] Moore M.J., Friedman J.A., Lewellyn E.B., Mantila S.M., Krych A.J., Ameenuddin S., Knight A.M., Lu L., Currier B.L., Spinner R.J. (2006). Multiple-channel scaffolds to promote spinal cord axon regeneration. Biomaterials.

[7] Chen X., Zhao Y., Li X., Xiao Z., Yao Y., Chu Y., Farkas B., Romano I., Brandi F., Dai J. (2018). Functional Multichannel Poly(Propylene Fumarate)-Collagen Scaffold with Collagen-Binding Neurotrophic Factor 3 Promotes Neural Regeneration After Transected Spinal Cord Injury. Adv. Healthc. Mater..

[8] Pawelec K.M., Hix J., Shapiro E.M., Sakamoto J. (2019). The mechanics of scaling-up multichannel scaffold technology for clinical nerve repair. J. Mech. Behav. Biomed. Mater..

[9] Papadimitriou L., Manganas P., Ranella A., Stratakis E. (2020). Biofabrication for neural tissue engineering applications. Mater. Today Bio.

[10] Liu Y., Sun S., Singha S., Cho M.R., Gordon R.J. (2004). 3D femtosecond laser patterning of collagen for directed cell attachment. Biomaterials.

[11] Sarig-Nadir O., Livnat N., Zajdman R., Shoham S., Seliktar D. (2009). Laser Photoablation of Guidance Microchannels into Hydrogels Directs Cell Growth in Three Dimensions. Biophys. J..

[12] De Paoli Lacerda S.H., Park J.J., Meuse C., Pristinski D., Becker M.L., Karim A., Douglas J.F. (2010). Interaction of gold nanoparticles with common human blood proteins. ACS Nano.

[13] Ren F., Yesildag C., Zhang Z., Lensen M. (2017). Surface Patterning of Gold Nanoparticles on PEG-Based Hydrogels to Control Cell Adhesion. Polymers.

[14] Tosa N., Bosson J., Vitrant G., Baldeck P., Stephan O. (2006). Fabrication of metallic nanowires by two-photon absorption. Sci. Study Res..

[15] Bellec M., Royon A., Bousquet B., Bourhis K., Treguer M., Cardinal T., Richardson M., Canioni L. (2009). Beat the diffraction limit in 3D direct laser writing in photosensitive glass. Opt. Express.

[16] Machida M., Niidome T., Onoe H., Heisterkamp A., Terakawa M. (2019). Spatially-targeted laser fabrication of multi-metal microstructures inside a hydrogel. Opt. Express.

[17] Fan C.H., Sun J., Longtin J.P. (2002). Breakdown threshold and localized electron density in water induced by ultrashort laser pulses. J. Appl. Phys..

[18] Schaffer C.B., Nishimura N., Glezer E.N., Kim A.M.-T., Mazur E. (2002). Dynamics of femtosecond laser-induced breakdown in water from femtoseconds to microseconds. Opt. Express.

[19] Noack J., Vogel A. (1999). Laser-induced plasma formation in water at nanosecond to femtosecond time scales: Calculation of thresholds, absorption coefficients, and energy density. IEEE J. Quantum Electron..

[20] Pradhan S., Keller K.A., Sperduto J.L., Slater J.H. (2017). Fundamentals of Laser-Based Hydrogel Degradation and Applications in Cell and Tissue Engineering. Adv. Healthc. Mater..

[21] Kang S., Vora K., Mazur E. (2015). One-step direct-laser metal writing of sub100nm 3D silver nanostructures in a gelatin matrix. Nanotechnology.

[22] Terakawa M., Torres-Mapa M.L., Takami A., Heinemann D., Nedyalkov N.N., Nakajima Y., Hördt A., Ripken T., Heisterkamp A. (2016). Femtosecond laser direct writing of metal microstructure in a stretchable poly(ethylene glycol) diacrylate (PEGDA) hydrogel. Opt. Lett..

[23] Izquierdo-Lorenzo I., Jradi S., Adam P.M. (2014). Direct laser writing of random Au nanoparticle three-dimensional structures for highly reproducible micro-SERS measurements. Rsc Adv..

[24] Tasche D., Weber M., Mrotzek J., Gerhard C., Wieneke S., Möbius W., Höfft O., Viöl W. (2020). In situ investigation of the formation kinematics of plasma-generated silver nanoparticles. Nanomaterials.

[25] Haiss W., Thanh N.T.K., Aveyard J., Fernig D.G. (2007). Determination of size and concentration of gold nanoparticles from UV-Vis spectra. Anal. Chem..

